# Supplementary figures and images for: Machine Learning of Stem Cell Identities From Single-Cell Expression Data via Regulatory Network Archetypes
Source: Front Genet. 2019 Jan 22;10:2. doi: 10.3389/fgene.2019.00002 (PMC6349820; doi:10.3389/fgene.2019.00002)

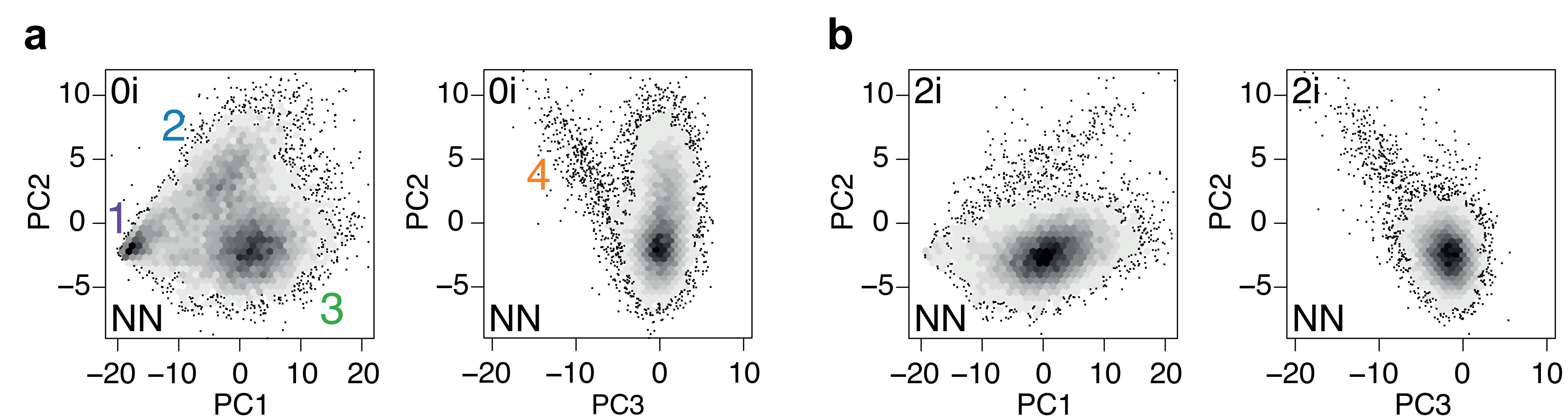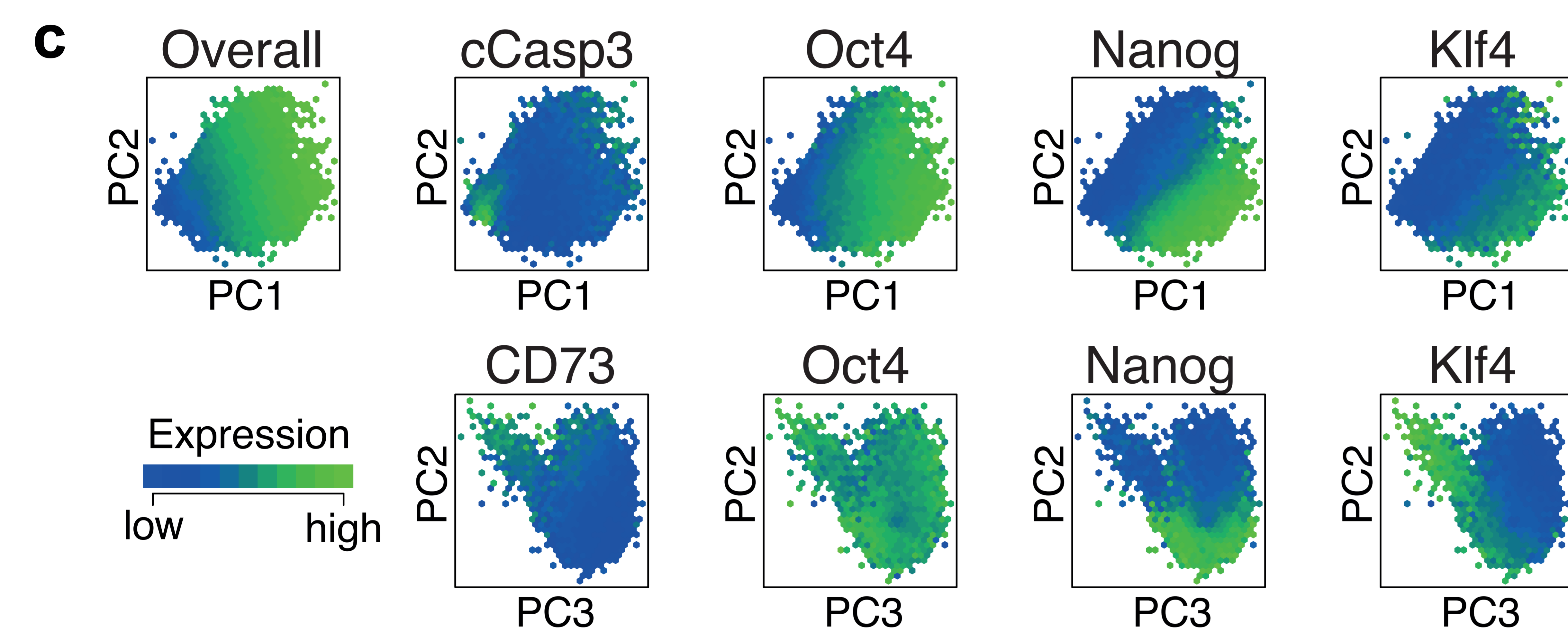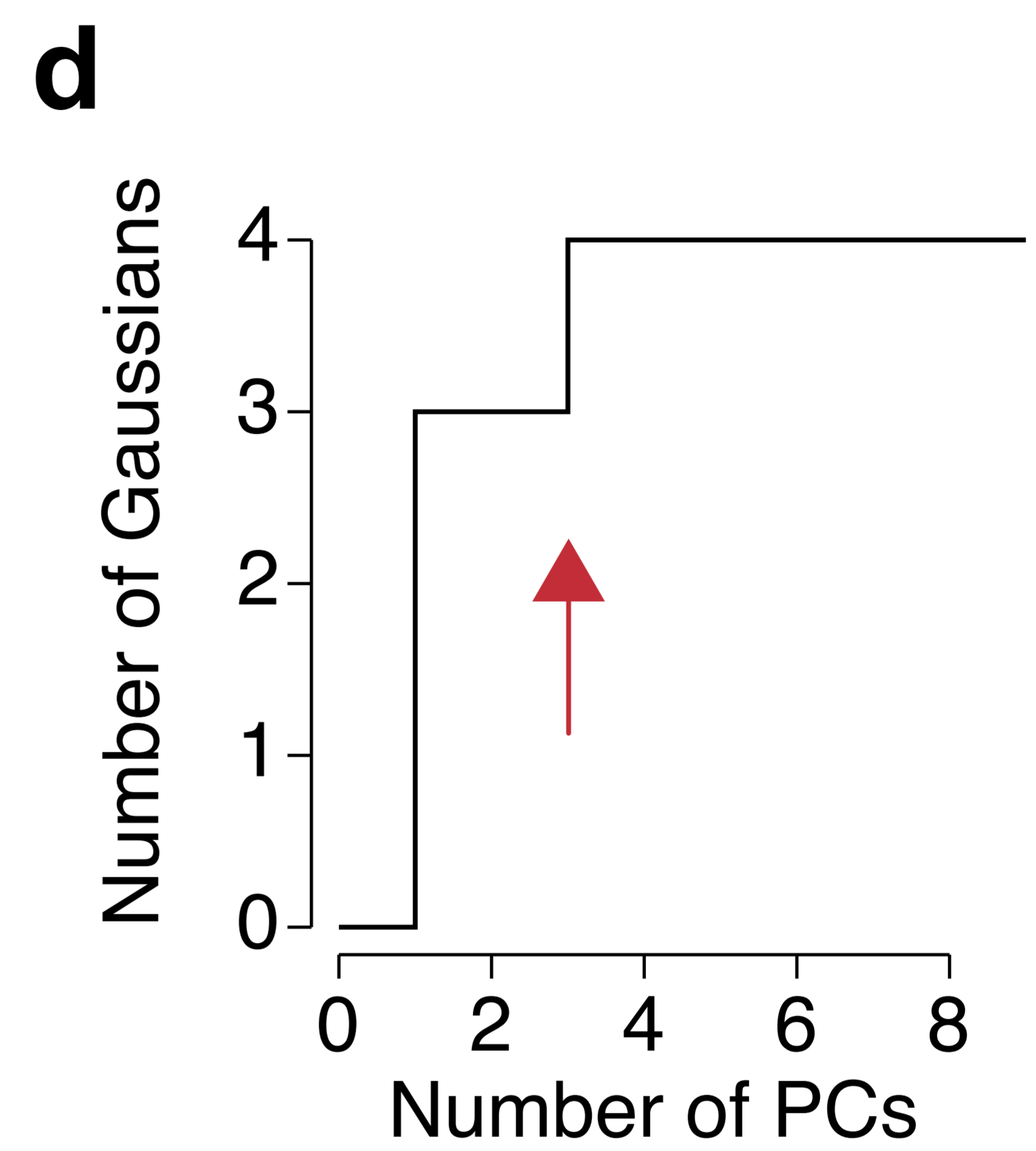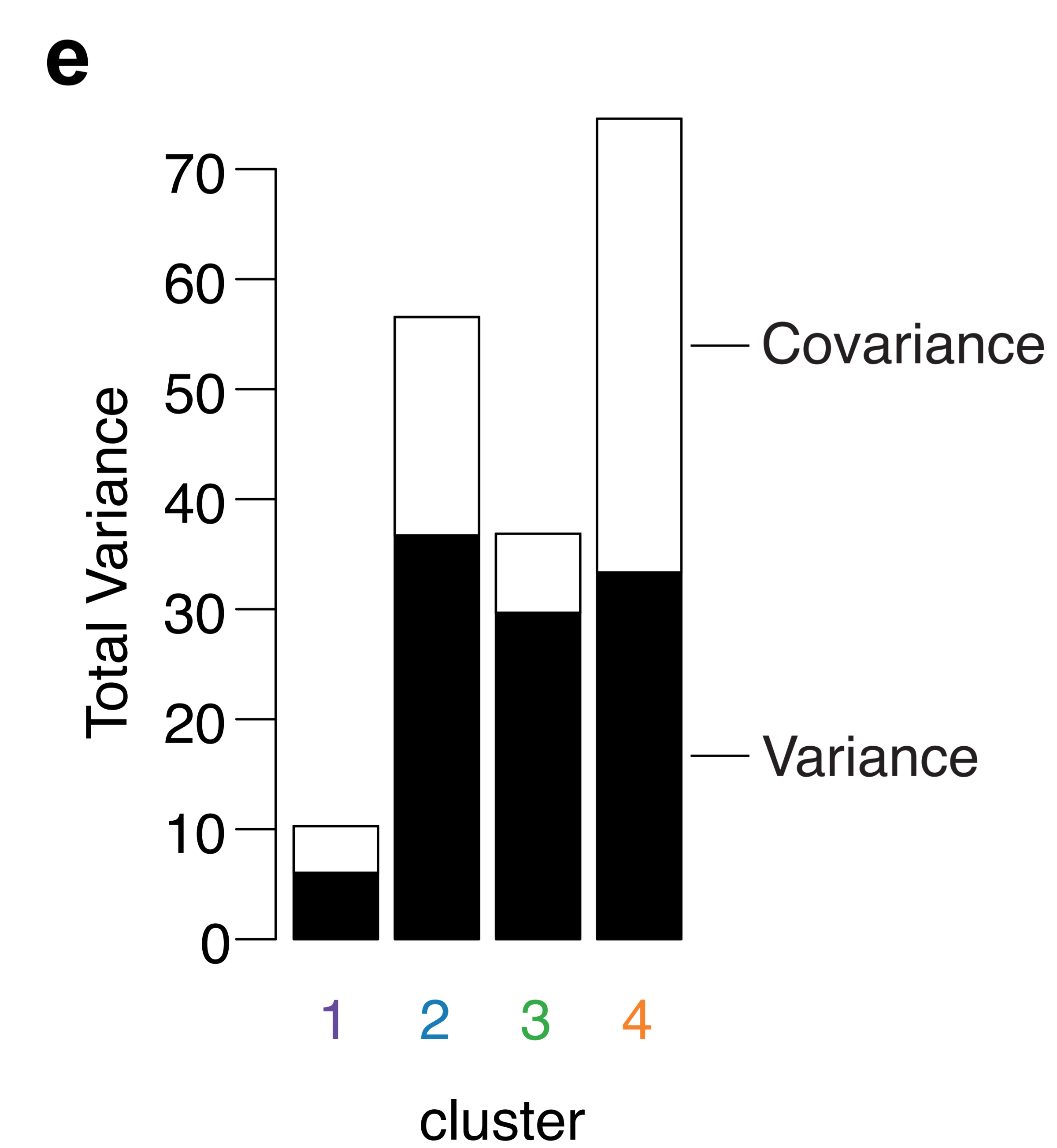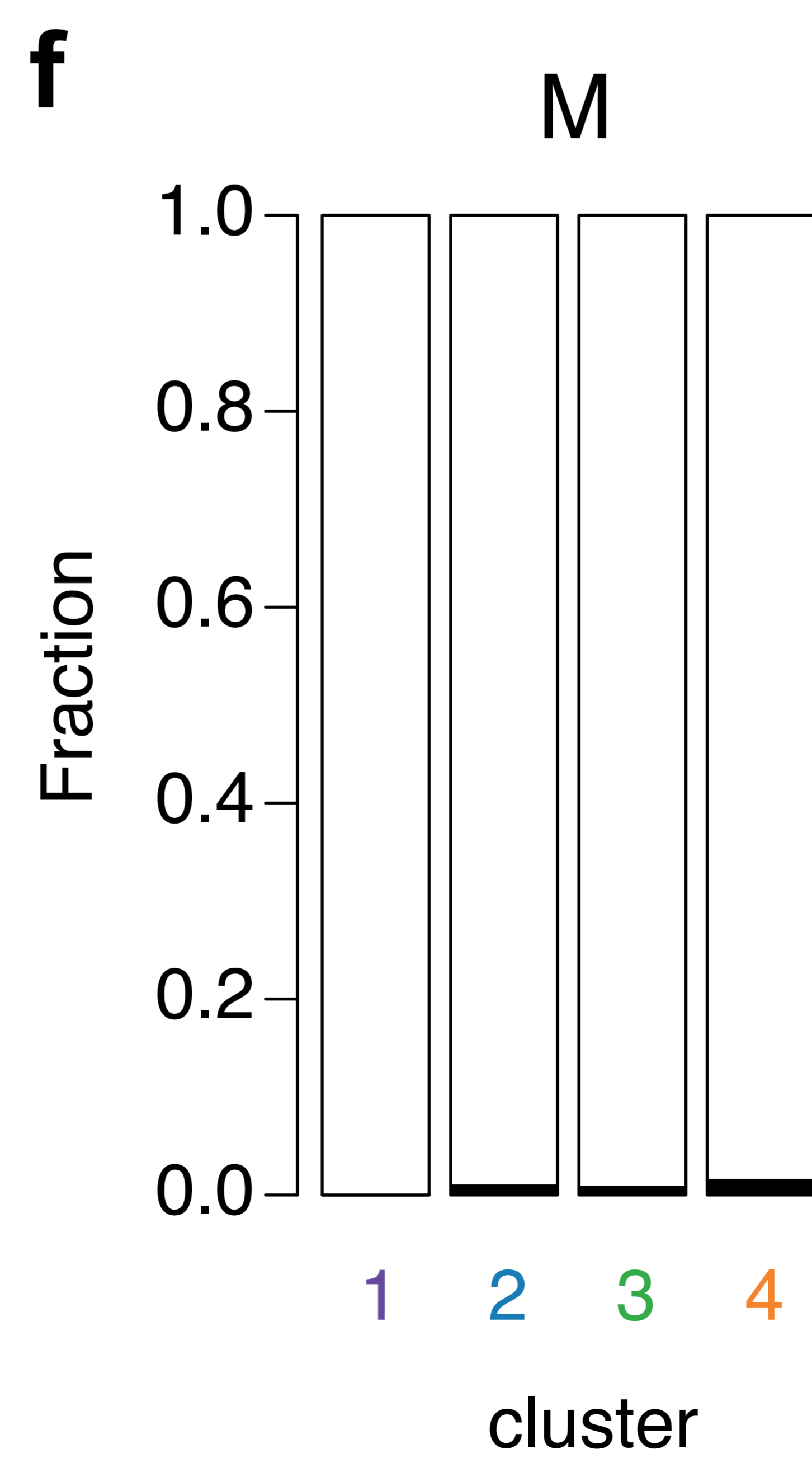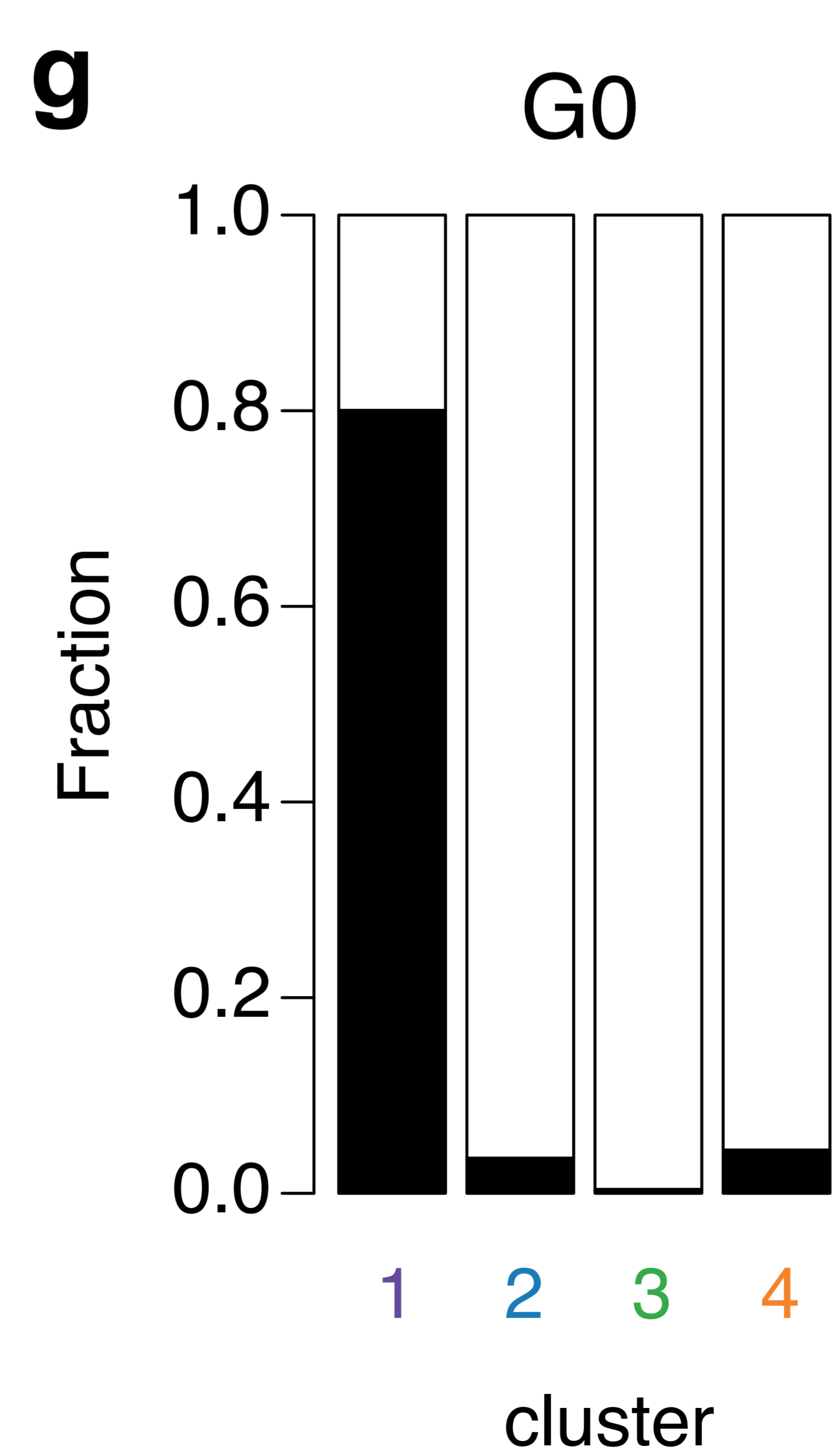

Supplement: Supplementary Figure 1 — Sub-states of regulatory network activity. (a–c) Projection of Nanog-Neo (NN) mESC onto the same principal component space derived from Nanog-GFP (NG) mESCs (shown in Figure 3). NN mESC display qualitatively the same population structure and corresponding node expression levels as NG mESCs. (d) Relationship between number of multivariate Gaussian distributions required to fully represent population structure, given the number of Principal Components used to represent network activity state. (e) Total variance/covariance within each sub-population (estimated from trace of the covariance matrix and the sum of the off diagonal elements of the covariance matrix for the respective fitted multivariate Gaussian models). (f) Fraction of cells of each cluster in M-phase of the cell cycle. sfig1 Fraction of cells of each cluster in G0-phase of the cell cycle. [file Image_1.pdf]
